# Rapidly Recurrent Retroperitoneal Cystic Lesion With Mass Effect in a Kidney Transplant Recipient: A Diagnostic and Management Challenge

**DOI:** 10.7759/cureus.105622

**Published:** 2026-03-21

**Authors:** Melissa L Barbosa, Joshua Samaniego, Renuka Chepuru, John P Findley, Kristen E Gebhardt, Emily Sestak, Samantha Betman, Oyedolamu Olaitan

**Affiliations:** 1 Surgery, Rush University Medical Center, Chicago, USA; 2 Pathology, Rush University Medical Center, Chicago, USA; 3 Transplant Nephrology, Rush University Medical Center, Chicago, USA

**Keywords:** cystic lesion, cystic retroperitoneal mass, cyst recurrence, immuno suppression, kidney transplant recipients, mass effect, renal cystic lesion

## Abstract

Complex cystic lesions arising from the kidney and adrenal gland are often found incidentally on cross-sectional imaging, as the majority are benign and asymptomatic. Large cystic lesions pose a diagnostic challenge, especially when initially simple and later demonstrating internal heterogeneity. This is consistent with radiological complex lesions, particularly in medically complex transplant patients, as there is no evidence-based consensus regarding when medical or surgical intervention is beneficial over regular monitoring. These challenges are amplified in solid-organ transplant recipients maintained on chronic immunosuppression, where altered immune surveillance may influence lesion behavior and clinical presentation. This report describes the case of a chronically immunosuppressed kidney-transplant recipient who was incidentally found to have an unusually progressive retroperitoneal lesion causing mass effect on the left native kidney, pancreas, stomach, and spleen. The lesion demonstrated progressive growth and increasing complexity on serial imaging and rapidly recurred following image-guided aspiration. Despite minimal early symptoms, the patient ultimately required an exploratory laparotomy with en bloc resection of the mass with left adrenalectomy and nephrectomy to fully excise a 20 cm cystic lesion. Pathologic evaluation was inconclusive despite extensive histologic and immunohistochemical analysis and review by multiple attending pathologists; no evidence of malignancy was identified. This case was characterized by rapid enlargement, recurrence after aspiration, and significant mass effect despite minimal symptoms. Such atypical behavior may be influenced by the patient’s immunosuppressive regimen, which is known to impair immune surveillance, inflammatory signaling, and reparative responses. This case highlights the need for careful surveillance and a low threshold for surgical intervention in transplant recipients with atypical or rapidly progressive cystic lesions.

## Introduction

Complex cystic lesions involving the adrenal gland and adjacent renal structures are uncommon, and their natural history is not well defined. Many of these lesions are benign and discovered incidentally, with only a minority demonstrating progressive enlargement requiring intervention [1-6]. Even less is known about how these lesions behave in the setting of solid-organ transplantation, where patients are maintained on chronic immunosuppression [1-4,6-13]. 

Immunosuppressive regimens, particularly those involving tacrolimus, mycophenolate, and prednisone, are known to alter the immune system, DNA repair pathways, and inflammatory signaling. Tacrolimus has been associated with disruptions to normal DNA repair processes and reduced detection of proliferating or dysregulated cells. Mycophenolate inhibits lymphocyte proliferation and may modify local inflammatory responses within tissues. Prednisone exerts broad anti-inflammatory and immunosuppressive effects that can blunt clinical manifestations of evolving pathology. Although these mechanisms have primarily been studied in the context of infection and malignancy risk, they may also potentially modify the evolution or clinical presentation of benign cystic lesions, without implying direct causation [11-13].

The interaction between cystic lesion behavior and chronic immunosuppression has not been described in the literature. In particular, no prior reports have examined whether post-transplant immunosuppressive therapy might contribute to rapid cyst enlargement, recurrent fluid accumulation, or unusually muted symptomatology. 

Here, we present a case of a kidney-transplant recipient on tacrolimus, mycophenolate, and prednisone who developed a rapidly enlarging and complex retroperitoneal cystic lesion that continued to enlarge despite aspiration. This report highlights the diagnostic challenges, potential underlying mechanisms behind the rapid growth and minimal symptoms, as well as the clinical implications of managing complex cystic lesions in chronically immunosuppressed individuals. 

## Case presentation

Patient background and clinical course 

A 49-year-old male patient with a history of focal segmental glomerulosclerosis (FSGS) was incidentally found on renal ultrasound to have a left-sided cyst adjacent to the left native kidney measuring 8.9 cm during pre-transplant imaging. Longitudinal imaging demonstrated that the lesion, initially simple, showed interval enlargement, internal heterogeneity, and mass effect on adjacent organs over several years, reported radiologically as a complex cystic lesion. He remained asymptomatic over the next few years with plans to monitor this lesion with serial imaging per our standard surveillance protocol for renal cysts.

The patient underwent a deceased-donor renal transplant (DDRT) and received induction immunosuppression with solumedrol and thymoglobulin, followed by maintenance immunosuppression with tacrolimus, mycophenolate, and prednisone. Following DDRT, the patient continued to have an expected post-transplant course, notably absent of any concerning clinical signs or symptoms related to the renal cyst.

Three years after his initial ultrasound, he underwent a surveillance ultrasound, which revealed a complex cystic lesion involving the left adrenal gland and native kidney, now measuring 14 x 13 x 13 cm. Subsequent MRI revealed progressive growth of the cyst measuring 14 x 12.7 x 13.2 cm, complicated by new mass effect in the retroperitoneum. 

Due to the unclear etiology of the complex cyst, an endoscopic ultrasound-guided aspiration was performed, which yielded 550 cc of cloudy, serous fluid. Cytology was nondiagnostic, and the anatomic origin of the cyst remained unclear. The aspirate contained blood products, and several weeks following the procedure, the patient was noted to have a significant decline in hemoglobin on routine laboratory evaluation. After discussion with interventional gastroenterology, repeat cross-sectional imaging was recommended.

**Figure 1:**
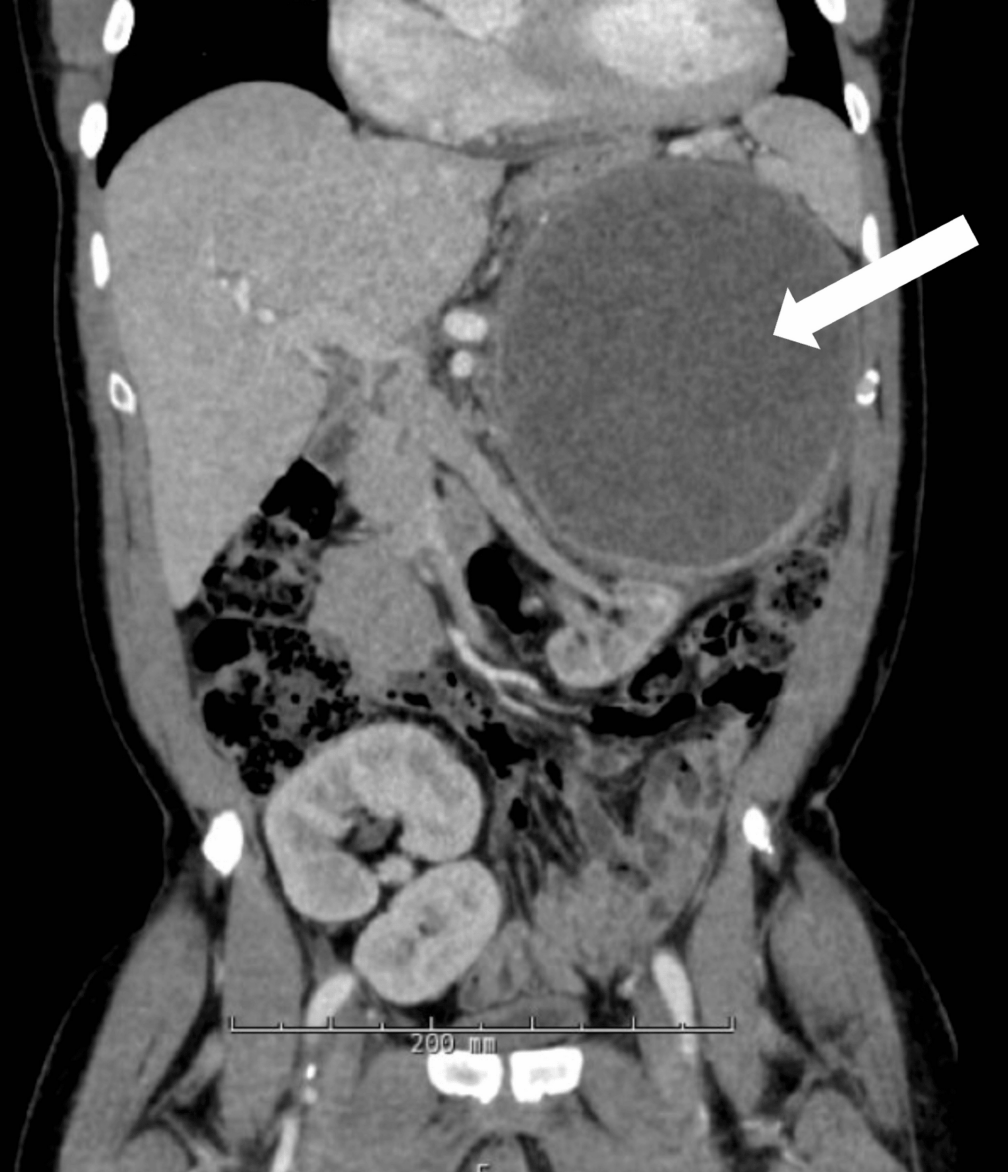
CT scan demonstrating displacement of the left native kidney with its vasculature, as well as compression of the pancreas, spleen, and stomach. White arrow indicates a cystic lesion with internal heterogeneity and mass effect, meeting the criteria for a complex cyst, causing displacement of the left native kidney, pancreas, spleen, and stomach

On repeat CT imaging (Figure 1), obtained as follow-up after aspiration and in the setting of hemoglobin decline, the cyst was noted to have continued to expand despite recent aspiration, increasing to 17.0 × 14.5 × 16.7 cm. At the time of repeat imaging, the patient remained largely asymptomatic, which was notable given the size of the lesion. 

Over the following three months, the patient developed progressive left-sided abdominal and flank pain, worsened with prolonged standing but without limitations in oral intake. In light of the rapid regrowth, increasing complexity, and evolving symptoms in the context of chronic immunosuppression, surgical intervention was recommended for definitive diagnosis and management.

Surgical, gross, and microscopic findings

**Figure 2:**
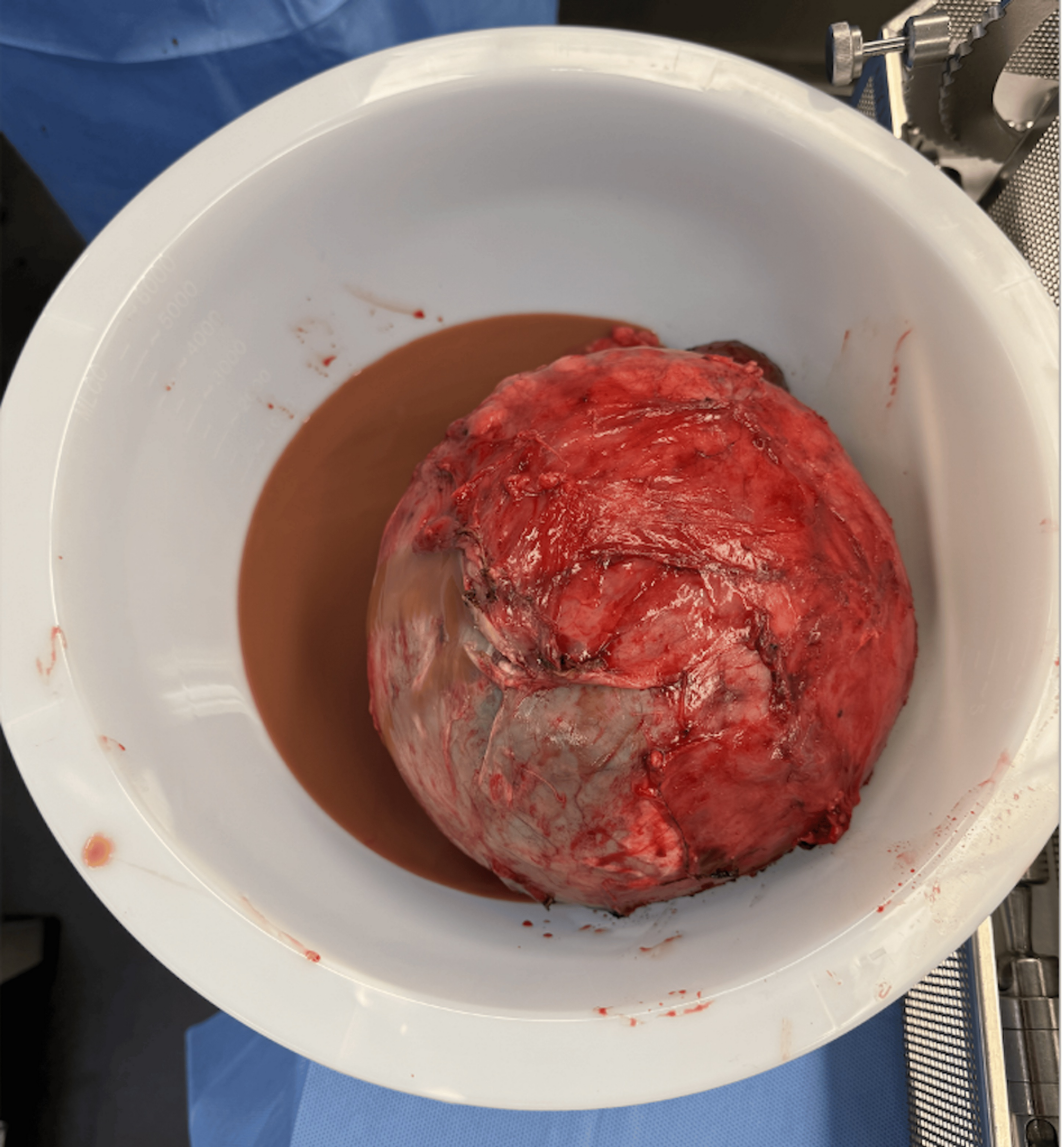
The excised large cystic lesion and associated purulent fluid.

The patient was taken for an exploratory laparotomy where an approximately 25 cm cystic mass (Figure 2), displacing both the left adrenal gland and native left kidney, was excised. This mass appeared separate from the stomach, pancreas, and spleen. A small amount of purulent-appearing fluid was encountered intraoperatively. The patient tolerated the procedure well, and his postoperative course was uneventful. The purulent-appearing fluid from the surgery later grew pan-sensitive *Streptococcus mitis*. While this organism is typically an oral commensal and contamination is possible, it was collected from purulent-appearing fluid under sterile conditions. The patient was seen by Infectious Disease and was recommended a course of antibiotics.

**Figure 3:**
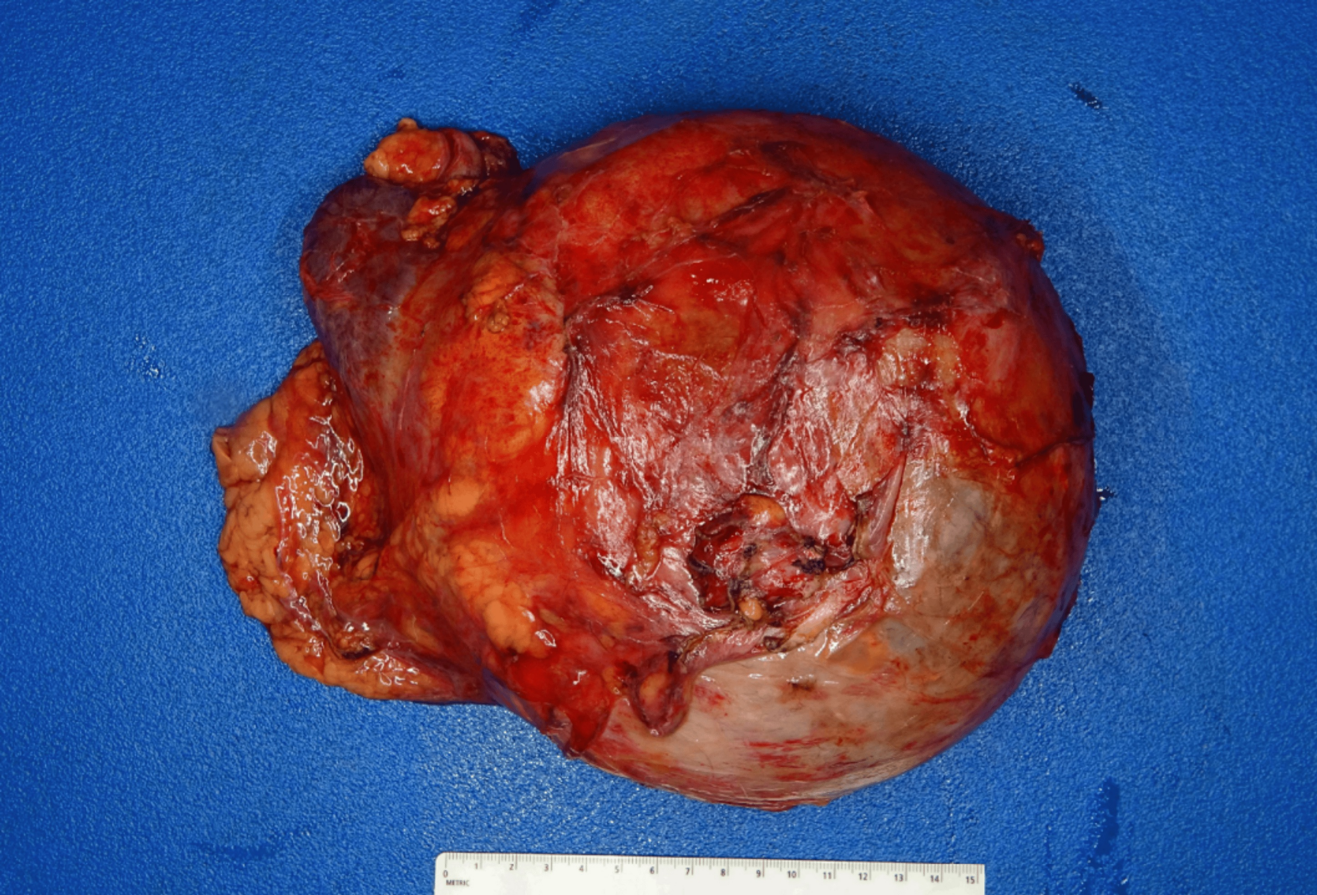
Gross image of giant retroperitoneal cyst (Reference ruler 15 cm).

**Figure 4:**
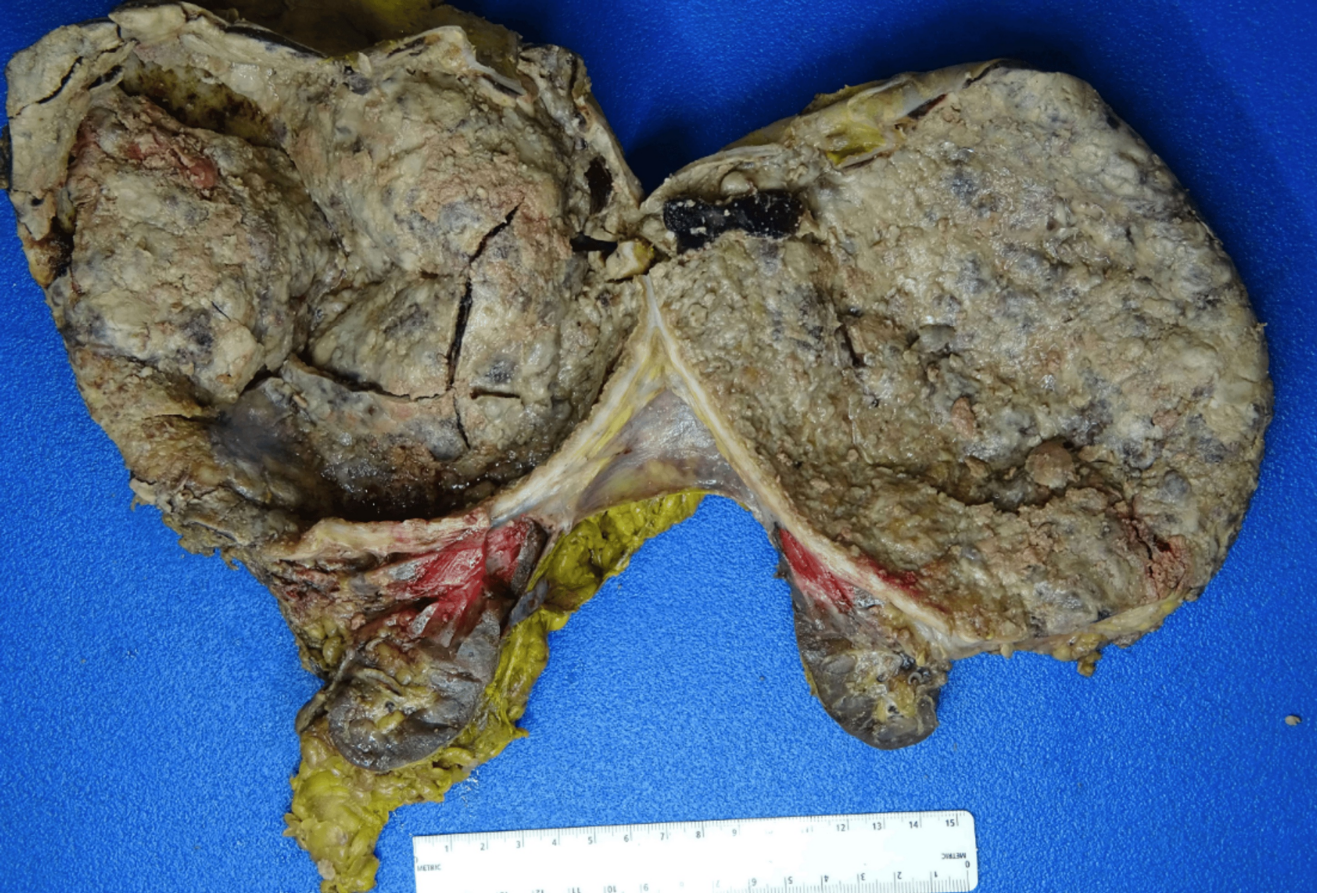
Gross image of giant adrenal cyst, bivalved (Reference ruler 15 cm).

The excised specimen included segments of the left adrenal gland, native left kidney, and distended left ureter. The cyst had a smooth surface and measured 27.8 cm at its greatest dimension, with a solid region along the superior cyst wall corresponding to adrenal tissue (1.7 cm). Upon bisection, copious hemorrhagic brown necrotic fluid was released, revealing a layer of necrotic crust along the internal surface of the cyst. No definitive mass-forming lesions were identified. The gross features of the intact and bivalved cyst are shown in Figure 3 and Figure 4, respectively.

The renal parenchyma, ureter, adrenal gland, and cyst wall were extensively sampled and analyzed microscopically to reveal benign adrenal gland tissue, identified by SF-1 and Melan-A immunohistochemistry, renal parenchyma with signs of kidney failure, benign urothelial tissue, and necrotic debris lining a fibrotic cyst wall with no definitive epithelial lining. Despite extensive sampling and immunohistochemistry (vimentin, CD10, CAIX, CD61, HMB-45, CD31, and chromogranin A), the origin of the cyst could not be definitively determined as renal versus adrenal, and no clear epithelial lining was identified.

**Figure 5:**
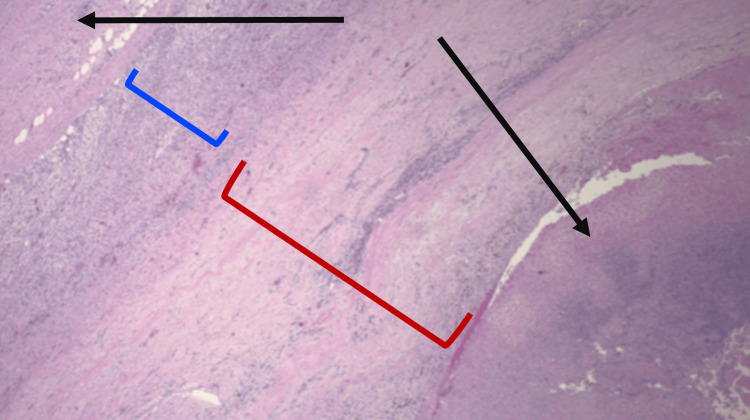
Magnified slide (4x) of adrenal tissue using H&E staining demonstrating thick fibrotic cyst wall and necrotic debris. Black arrows indicates areas of necrosis; Blue bracket indicates area of adrenal gland tissue with intermixed histiocytes; Red bracket indicates area of cyst wall.

**Figure 6:**
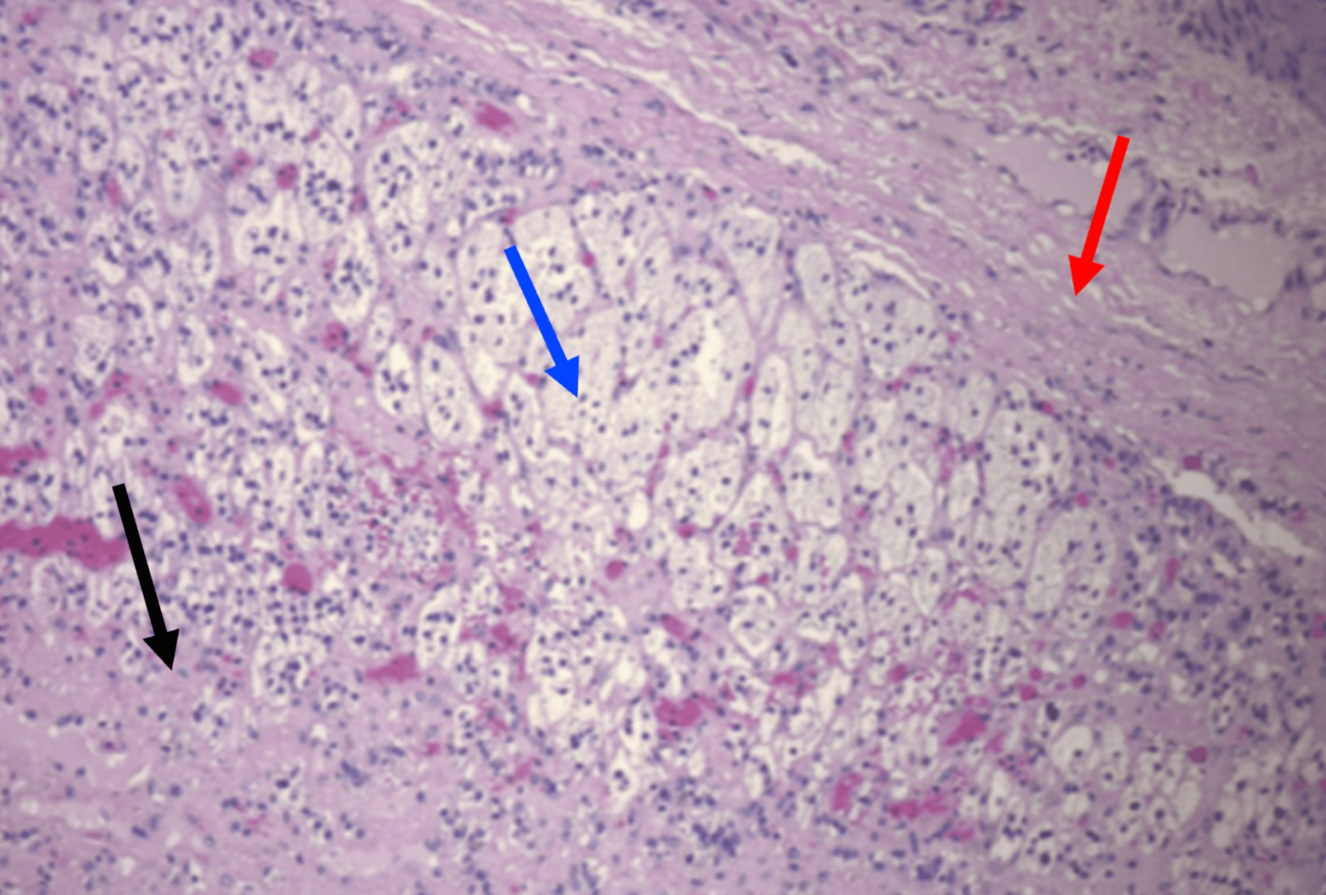
Magnified slide (10x) of adrenal tissue using H&E staining. Black arrows indicates areas of necrosis; Blue bracket indicates area of adrenal gland tissue with intermixed histiocytes; Red bracket indicates area of cyst wall.

Microscopy revealed benign adrenal tissue, renal parenchyma with features of kidney failure, benign urothelial tissue, and necrotic debris lining a fibrotic cyst wall. Representative histologic findings are shown in Figures 5, 6. 

## Discussion

This case illustrates an unusually aggressive course of a retroperitoneal cystic lesion in a patient maintained on chronic post-kidney transplant immunosuppression. Although complex cystic lesions in the retroperitoneum are typically benign and slow-growing [1-6], this patient’s lesion demonstrated rapid enlargement over five months, recurrence after aspiration, and significant mass effect, yet produced relatively muted symptoms, features that are atypical in immunocompetent individuals. This combination of rapid progression and muted clinical presentation is highly atypical in immunocompetent individuals but is plausibly influenced by the patient’s immunosuppressive regimen [1-4,6-11,13]. 

The lesion may represent a pre-existing simple renal cyst that subsequently underwent intracystic hemorrhage, resulting in chronic inflammatory changes and the observed complex appearance on imaging. Extensive necrosis and fibrotic debris prevented definitive determination of the cyst’s origin as renal versus adrenal, even after comprehensive histologic and immunohistochemical analysis. This uncertainty broadens the differential diagnosis to include adrenal pseudocysts, hemorrhagic renal cysts, endothelial cysts, and other rare retroperitoneal cystic lesions. 

While causation cannot be established, chronic immunosuppressive therapy may act as a modifying factor, contributing to atypical lesion behavior. The biological mechanisms underlying these risks, including dampened immune surveillance, reduced inflammatory signaling, and altered DNA repair responses, may also impact the growth of benign cysts [11,13]. Evidence from transplant oncology literature supports that immunosuppressive medications can promote unregulated growth of malignant masses, although no studies have specifically examined their effects on benign cystic lesions. 

Fluid obtained from the cyst at the time of surgery grew *S. mitis*. Although typically an oral commensal and possible laboratory contaminant, the sterile collection and purulent appearance suggest secondary colonization or infection, which may have been promoted by immunosuppression. Targeted postoperative antibiotic therapy was administered without further complications. These findings support a pathophysiologic process in which impaired inflammatory clearance in the setting of chronic immunosuppression may contribute to increasing cyst complexity and rapid growth, while simultaneously blunting the expected clinical manifestations of infection or inflammation [13].

The gross and microscopic pathology of the specimen demonstrated notable similarities to a benign dermoid cyst. Although the current specimen showed no epithelial differentiation, and the origin of this cystic lesion could not be definitely established, the previously noted association between the development of epidermoid cysts in renal transplant patients on immunosuppressive therapy could suggest an unexplored pathophysiologic association between immunosuppression and unregulated epithelial growth in solid organs [11-13]. 

Another key challenge in this case was the diagnostic uncertainty of this large cystic lesion, as it is often difficult to distinguish complex renal, adrenal, and pancreatic lesions on imaging [1-4,6-10]. In this situation, aspiration and imaging were non-diagnostic, and the decision to proceed with surgical exploration was ultimately justified by the lesion’s rapid growth and mass effect on other organs. 

Due to extensive necrosis noted on gross pathology, it was impossible to distinguish whether the cyst had an epithelial lining, making it a true cyst, or whether it was a pseudocyst without an epithelial lining. It was also difficult to distinguish whether the cyst was of adrenal or renal origin because the adrenal gland was fused to the cyst, while the cyst was fused to the upper pole and interpolar region of the kidney secondary to chronic inflammation. 

No signs of malignancy were identified by gross, histologic, and immunohistochemical examination, but malignancy cannot be definitively ruled out in this case due to the necrotic nature of the specimen. The differential diagnosis for this case includes, but is not limited to, apoplectic adrenal cortical adenoma with secondary cystic and hemorrhagic change, adrenal pseudocyst (hemorrhagic or inflammatory type), endothelial (vascular) cyst, epithelial adrenal cyst, or infectious cyst. 

The delayed clinical presentation in this patient is likely attributable, in part, to the cyst association with the nonfunctioning native kidney. Had a lesion of a comparable size developed within the functioning renal allograft, earlier symptomatology or laboratory abnormalities would be expected, leading to an earlier diagnosis. Because the native kidney provided no physiologic contribution to the patient’s renal function, the cystic lesion was able to enlarge substantially and exert mass effect on adjacent organs while remaining clinically silent, thereby delaying recognition until the cyst had reached an advanced size.

## Conclusions

This case raises an important and previously unreported consideration: chronic immunosuppression may influence the growth dynamics and symptomatic profile of retroperitoneal cystic lesions. Although causation cannot be established, the rapid growth, recurrence after aspiration, and muted symptoms together highlight a plausible and clinically relevant association. Careful monitoring and a low threshold for surgical intervention, both for diagnostic and therapeutic purposes, should be considered in transplant recipients with atypical cystic lesions. 
